# Clinical Application of Liquid Biopsy in Non-Hodgkin Lymphoma

**DOI:** 10.3389/fonc.2021.658234

**Published:** 2021-03-18

**Authors:** Liwei Lv, Yuanbo Liu

**Affiliations:** Department of Hematology, Beijing Tiantan Hospital, Capital Medical University, Beijing, China

**Keywords:** non-Hodgkin lymphoma, diffuse large B-cell lymphoma, primary central nervous system lymphoma, liquid biopsies, cell-free DNA, circulating tumor DNA, microRNA, tumor-derived exosomes

## Abstract

Non-Hodgkin lymphoma (NHL) is a common type of hematological malignant tumor, composed of multiple subtypes that originate from B lymphocytes, T lymphocytes, and natural killer cells. A diagnosis of NHL depends on the results of a pathology examination, which requires an invasive tissue biopsy. However, due to their invasive nature, tissue biopsies have many limitations in clinical applications, especially in terms of evaluating the therapeutic response and monitoring tumor progression. To overcome these limitations of traditional tissue biopsies, a technique known as “liquid biopsies” (LBs) was proposed. LBs refer to noninvasive examinations that can provide biological tumor data for analysis. Many studies have shown that LBs can be broadly applied to the diagnosis, treatment, prognosis, and monitoring of NHL. This article will briefly review various LB methods that aim to improve NHL management, including the evaluation of cell-free DNA/circulating tumor DNA, microRNA, and tumor-derived exosomes extracted from peripheral blood in NHL.

## Introduction

Non-Hodgkin lymphoma (NHL) is a common type of hematological malignant tumor, composed of multiple subtypes that originate from B lymphocytes, T lymphocytes, and natural killer (NK) cells, including diffuse large B cell lymphoma (DLBCL), follicular lymphoma (FL), T cell lymphoma (TCL), and NK-T cell lymphoma (NKTCL). The most common NHL subtype is DLBCL, which is a group of heterogeneous tumors (1). This heterogeneity is reflected by significant differences in the responses of these tumors to the standard first-line treatment strategy, known as R-CHOP (rituximab, cyclophosphamide, doxorubicin hydrochloride, oncovin, and prednisone). Approximately 20%–50% of patients experience relapse or develop drug-resistance after treatment (2). The 5-year survival rates for DLBCL reported for the United States and Europe are 63.8% and 55.4%, respectively (2). Primary central nervous system lymphoma (PCNSL) is a rare type of primary extranodal NHL that originates in the brain parenchyma, cranial nerves, leptomeninges, eyes, or spinal cord, without affecting other sites. In greater than 90% of PCNSL, the pathology is consistent with DLBCL. With the application of high-dose methotrexate-based combination chemotherapy, the median overall survival rate of PCNSL increased from 12.5 months in the 1970s to 26 months in the 2010s (3). Similar to systemic DLBCL, approximately 50% of PCNSL patients relapse after treatment (4), and 10% to 15% of patients are primarily refractory (5). In cases of refractory or relapsed disease, the prognosis is typically poor.

**Figure 1:**
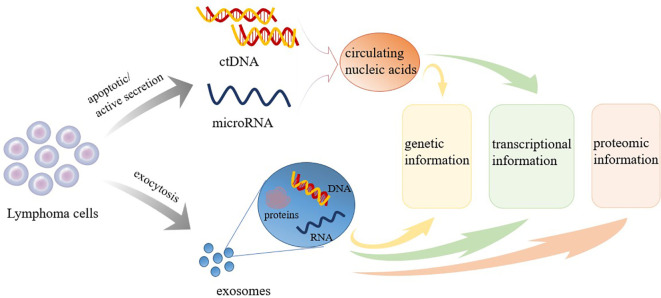
The value of liquid biopsy. ctDNA, circulating tumor DNA.

**Figure 2:**
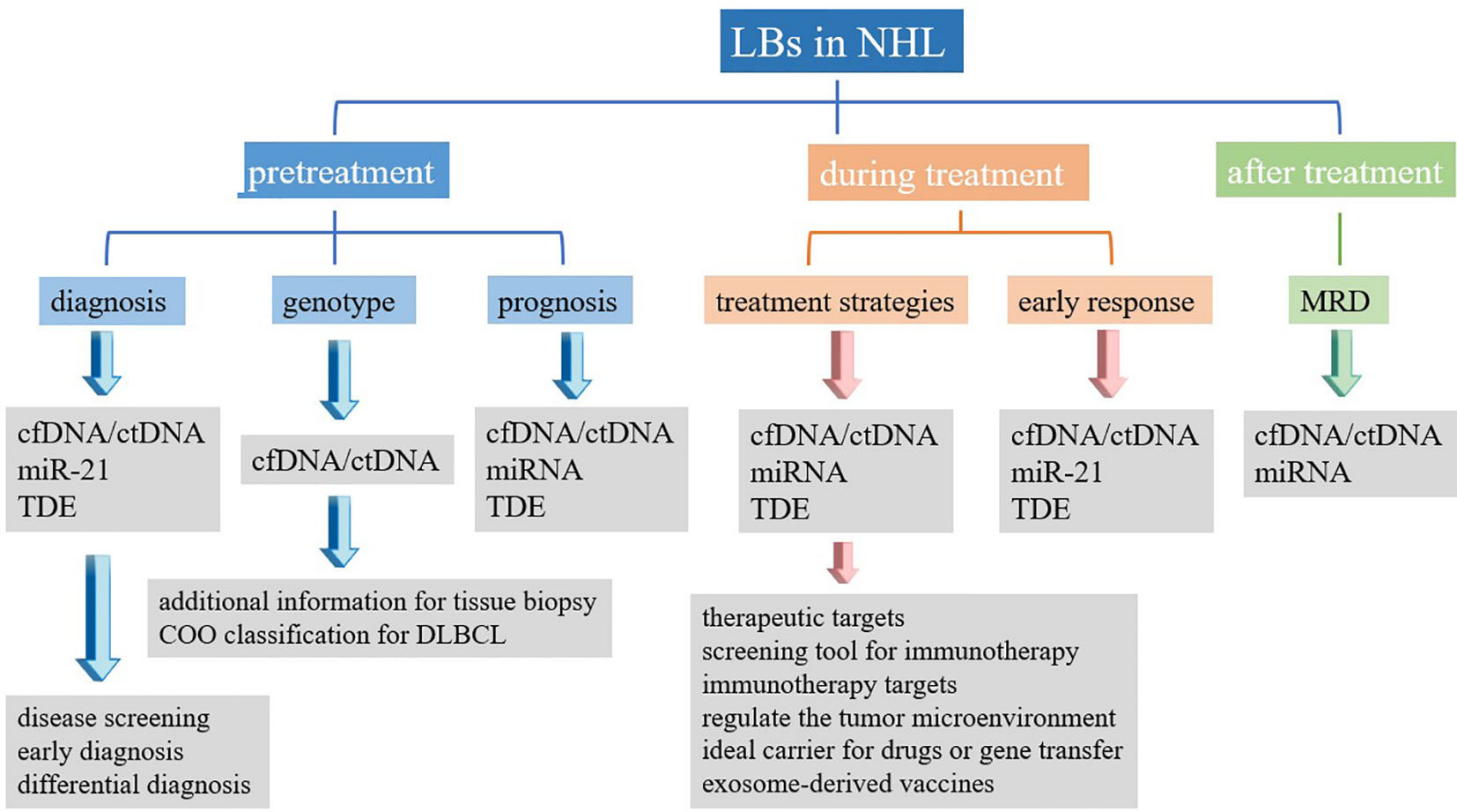
Summary of clinical application of liquid biopsy in non-Hodgkin’s lymphoma. LBs, liquid biopsies; NHL, non-Hodgkin’s lymphoma; cfDNA, cell-free DNA; ctDNA, circulating tumor DNA; miRNA, microRNA; TDE, tumor-derived exosomes; MRD, minimal residual disease.

Regardless of the NHL subtype identified, the current gold standard for diagnosis remains the pathological examination of the affected tissue, which is typically obtained through surgical resection or lymph node puncture. However, tissue biopsies are invasive examinations that can only reflect the characteristics of tumors statically and locally at the biopsy location. Biopsies cannot be used for the dynamic monitoring of tumors during treatment and follow-up (6). Many disadvantages, including the risks associated with tissue biopsy (bleeding, infection, functional impairment, etc.), the complexity of obtaining biopsy samples, and difficulties associated with reproducibility, have significantly hampered the development of clinical applications for tumor evaluation and prediction. To overcome these barriers, the concept of liquid biopsies (LBs) has been introduced. LBs are characterized by being noninvasive, allowing for the convenient and continuous collection and dynamic monitoring of samples that can provide information regarding disease progression (7, 8). LBs are considered to serve as beneficial supplements to tissue biopsies. LBs can be used for screening and early detection, assessing prognosis, real-time monitoring of response to treatment, guiding treatment, identifying treatment targets, and detecting early recurrence (8). Various forms of LBs are currently widely studied, including circulating tumor cells (CTCs), circulating blood nucleic acids, tumor-derived exosomes (TDE), and tumor-educated platelets (9). Initially, studies of LB focused on CTCs, but recent research focus has shifted to circulating nucleic acids, which are easier to obtain and analyze (7). This article primarily reviews the clinical applications of various types of LBs, including cell-free DNA (cfDNA)/circulating tumor DNA (ctDNA), microRNA (miRNA), and TDE extracted from peripheral blood, in the assessment and treatment of NHL. cfDNA/ctDNA sheds into blood from tumor cells *via* apoptosis, necrosis or through an active process (**Figure 1**). Molecular aberrations in tumor tissues, such as point mutations, insertions, deletions, and alterations in DNA methylation can be detected in cfDNA/ctDNA. Tumor cells can also shed different classes of RNA into the bloodstream. Among RNAs, miRNA is the most frequently investigated RNA species as biomarkers (**Figure 1**). The TDEs are produced by tumor cells and secreted into the bloodstream through exocytosis. TDE contents include proteins, nucleic acids, lipids and metabolites (**Figure 1**). Therefore, LBs can yield data regarding genetic, transcriptional, and proteomic changes useful for diagnosis, prognosis, and therapy of NHL (**Figure 1**). LB can be used for screening and early detection, assessing prognosis, real-time monitoring of response to treatment, guiding treatment, identifying treatment targets, and detecting early recurrence of NHL (**Figure 2**). A comprehensive understanding of the clinical application value of LBs will help clinicians better manage NHL patients, and achieve the purpose of improving treatment effects and improving prognosis.

## Characteristics and Detection Methods Used in LBs

Following apoptosis or necrosis, both normal cells and tumor cells release nucleic acids (DNA, mRNA, and miRNA) into bodily fluids, such as the peripheral blood, cerebrospinal fluid, and urine (10, 11). Non-cell-bound DNA fragments found in the circulatory system are referred to as cfDNA, which typically consists of both normal DNA and ctDNA (12). The origins of the tumor portion of cfDNA may be apoptosis or necrosis, the lysis of CTCs, or the release of DNA from tumor cells into the circulation (13). Previous research has confirmed that cfDNA typically appears as fragments, with sizes that peak at 166–167 bp, and ctDNA is usually even shorter than normal cfDNA (14, 15). The proportion of ctDNA in cfDNA can range from 3% to 93% (16). Current methods for detecting circulating DNA include polymerase chain reaction (PCR) and next-generation sequencing (NGS, **Table 1**). The detection limit of PCR is 10^−5^ (17), and the two most commonly used PCR-based detection techniques are real-time quantitative PCR and droplet digital PCR (18, 19). Epigenetic changes can be detected by pyrosequencing (20). In contrast to PCR, which only targets specific DNA sequences, the detection range for NGS is broader because it targets the entire genome or the entire exome (17). Compared with PCR, the detection rate for NGS increase to 10^−6^ (21). The two platforms commonly used in DLBCL research are noninvasive tumor immunoglobulin gene NGS (IgNGS) and cancer personalized profiling by deep sequencing (CAPP-Seq). The cfDNA/ctDNA can be used for cancer diagnosis, prognosis, and monitoring. However, cfDNA can also be upregulated by other conditions, such as infection, trauma, inflammation, transplantation, and autoimmune disorders (22), which can introduce uncertainties to the interpretation of cfDNA results.

**Table 1:** Detection methods used by liquid biopsy.

	Methods		Characteristics
cfDNA ctDNA	PCR	qRT-PCR ddPCR	Targets certain DNA sequences
	NGS	IgNGS CAPP-Seq	Higher sensitivity and wider coverage
miRNA	RT-PCR		High sensitivity and specificity
	NGS		Sequencing
	Microarray		High-throughput analysis Requires enough initial samples
TDE	Ultracentrifugation		Requires higher speeds and longer durations Less suitable for fluids with high viscosity
	Size-based techniques	Ultrafiltration	Faster than ultracentrifugation Protein contamination
		SEC	Pure isolates with intact physical characteristics
	PEG precipitation		Simple, low-cost, rapid Co-precipitated proteins
	Immunoaffinity enrichment		High-purity separation

cfDNA, cell-free DNA; ctDNA, circulating tumor DNA; TDE, tumor-derived exosomes; PCR, polymerase chain reaction; qRT-PCR, real-time quantitative PCR; ddPCR, droplet digital PCR; NGS, next-generation sequencing; CAPP-Seq, cancer personalized profiling by deep sequencing; RT-PCR, reverse transcription PCR; SEC, size exclusion chromatography; PEG, polyethylene glycol.

MicroRNAs are small, regulatory, non-coding RNAs that can negatively regulate gene expression at the post-transcriptional level by binding to target mRNA molecules, resulting in the increased degradation or translational inhibition of the targeted mRNA (23). Evidence has shown that tumor-related miRNAs can be detected in bodily fluids (such as serum or plasma) (24). As biomarkers, circulating miRNAs play an essential role in the diagnosis, typing, treatment response monitoring, and prognostic prediction of NHL. The detection of miRNA can be performed by reverse transcription PCR, NGS, and microarray techniques (25). At present, in most NHL studies, miRNA expression has been evaluated in serum.

Exosomes are a type of endosome-derived extracellular vesicle with a diameter ranging from 30 to 120 nm (2). Exosomes typically contain nucleic acids, proteins, lipids, and metabolites (26). Similar to cfDNA, exosomes can also be found in a variety of bodily fluids, including peripheral blood, cerebrospinal fluid, urine, saliva, and peritoneal fluid (27). Exosomes have been shown to participate in cell-to-cell communications and can affect the phenotypes of recipient cells (28). Exosomes play important roles during both physiological and pathological conditions, including the maintenance of cell homeostasis and the regulation of gene transcription, and exosomes have been shown to participate in the immune response and tumor progression (29). Exosomes differ depending on their cellular origins, and the contents and expression of exosomes secreted by healthy cells differ from those secreted by tumor cells. Therefore, TDEs represent excellent biomarkers for the diagnosis, prognosis, and management of NHL patients at multiple stages. Many methods can be used to isolate exosomes (**Table 1**). A commonly used method for exosome separation is differential ultracentrifugation; however, this method is not suitable for processing high-viscosity fluids due to the requirement for higher speeds and longer centrifugation durations (30). Density-gradient ultracentrifugation is similar to differential ultracentrifugation and can be used to obtain purer fractions; however, density-gradient ultracentrifugation requires long processing times and expensive equipment, which are disadvantages (31). Ultrafiltration and size exclusion chromatography can be used to separate exosomes according to size. The polyethylene glycol precipitation method is a relatively simple, low-cost, and rapid method that can produce high yields while retaining the biophysical properties of isolated exosomes. However, co-precipitated proteins can contaminate exosomes in ultrafiltration and polyethylene glycol precipitation method (32). The immunoaffinity enrichment method uses antibodies against extracellular membrane markers (usually the transmembrane proteins CD9, CD63, and CD81) to isolate exosomes from biological fluids (9). The immunoaffinity method can result in high-purity separation and has been proven to be a very effective method for the separation of exosomes. These various extraction methods are each associated with advantages and disadvantages, and different methods can be selected according to the aims of the experiment.

## LBs in Diagnosis

Although tissue biopsies are invasive, a pathological NHL diagnosis based on the histological examination of a tissue biopsy sample remains the “gold standard.” As a noninvasive test, cfDNA assessments are safer and more reproducible than tissue biopsies and are, therefore, likely to become a standardized detection method for disease screening and early diagnosis. Several studies have shown that cfDNA is increased in DLBCL compared with the normal control group, with a sensitivity of 70%–82.5% (33–35) and a specificity of 62.8%–94% (34–36). The median cfDNA concentration has varied across studies, ranging from 11.7–845 ng/ml in DLBCL, 12.4–662 ng/ml in NKTCL, and 12.9–942 ng/ml in TCL (33, 35–37). Currently, the most commonly used methods to detect cfDNA include various types of PCR. Commonly used methods to quantify cfDNA/ctDNA are Qubit for quantification (33, 37) and quantitative PCR (34–36). Although differences in the detection methods used have resulted in heterogeneity among the cfDNA concentrations reported by different studies, all of these studies have reached the conclusion that cfDNA concentrations in NHL cases are higher than those in normal controls. This finding suggests that cfDNA might be used as a diagnostic indicator. Sun et al. compared and analyzed the cfDNA concentrations of DLBCL and NKTCL patients and found that the cfDNA concentration in NKTCL patients was significantly higher than that in DLBCL patients (19.6 ng/ml vs. 11.7 ng/ml, p = 0.027) (37). This result suggested that cfDNA also has the potential to identify different types of NHL. However, studies have also demonstrated that cfDNA concentration can be elevated during many non-tumor diseases, such as sepsis, severe trauma, liver damage, and immune diseases (22). Therefore, the analysis and interpretation of cfDNA data may be challenging for both diagnosis and differential diagnosis.

At present, a variety of circulating miRNAs in DLBCL have been studied, resulting in the identification of greater than 80 miRNAs, to date. Nine specific miRNAs have been examined in multiple studies, including miR-21, miR-155, miR-210, miR-15a, miR-29c, miR-494, miR-34a, miR-145, and miR-379 (38–51). However, only two miRNAs have been confirmed to be elevated in DLBCL cases compared with their levels in normal individuals: miR-21 (38–40, 44, 46, 50, 51) and miR-155 (40–42, 44, 46, 49). Moreover, the study by Chen et al. showed that the level of miR-21 in the activated B cell-like (ABC) group was higher than that of the germinal center B cell-like (GCB) group (39). A study by Eis et al. of DLBCL cell lines found that the miR-155 level in DLBCL with an ABC phenotype was higher than in cells with the GCB phenotype (42). Interestingly, Caivano et al. studied miR-155 in extracellular vesicles from 5 DLBCL patients and found that miR-155 level in extracellular vesicles in the DLBCL group did not differ from that in the normal group (52). However, the number of cases examined in this study was relatively limited. Two other miRNAs have been confirmed to be downregulated in DLBCL cases compared with normal controls, miR-145 (46, 47) and miR-379 (47, 48), but each of these has only been evaluated in two studies. Beheshti’s team identified 10 related circulating miRNAs (let-7b, let-7c, miR-10b, miR-130a, miR-155, miR27a, miR-24, miR-18a, miR15a, and miR-497) in the DLBCL mouse model (53). Since then, the team has shown that 5 multi-miRNA signature (let-7b, let-7c, miR-18a, miR-24, and miR-15a) can be used to differentiate DLBC from control groups with high sensitivity and specificity (90% and 94%, respectively) (45). In addition to miRNAs that are upregulated in DLBCL, recent studies have identified some miRNAs that are downregulated in DLBCL, such as miRNA-16-1 (54), miR-425, miR-141, miR-197, miR-345, miR-424, miR-128, and miR-122 (47). Unfortunately, none of these miRNAs has been verified repeatedly. The peripheral circulatory system of DLBCL patients is rich in miRNAs, which carry a lot of information. Currently, only miR-21 is recognized as having the potential to become a diagnostic marker for DLBCL. In addition to DLBCL, Guo et al. compared 79 NKTCL patients with 37 normal plasma samples to examine miR-221 and found that the miR-221 level in the NKTCL group was significantly higher than that in the normal group (55). However, current research on circulating miRNAs primarily focuses on DLBCL, with few studies on other NHL subtypes; therefore, many areas remain to be explored regarding circulating miRNAs.

Although exosomes can contain much information, including nucleic acids and proteins, research regarding exosomes in NHL remains scarce. Xiao et al. found that the expression level of the serum exosome miR-451a was significantly reduced in the DLBCL group compared with that in the control group, and serum exosome miR-451a levels (p < 0.01) had a moderate diagnostic efficiency for DLBCL (56). In PCNSL, Gállego et al. showed that RNU6-1 levels were significantly lower in serum exosomes from PCNSL patients than in those from glioblastoma patients (18.1 copies/20 µL vs. 412 copies/20 µL; P = 0.004) (57). The expression of RNU6-1 in serum exosomes may be used for the differential diagnosis between PCNSL and glioblastoma (57). However, so few related studies exist that the exact contribution of TDE analysis to the diagnosis of NHL is difficult to determine. The value of TDE for the diagnosis and differential diagnosis of NHL requires additional research and further exploration.

## LBs in Tumor Genotyping and Cell-of-Origin Classification

In 2017, Rossi et al. detected DLBCL-related mutations in cfDNA among patients with DLBCL, and the results showed that genetic variants with allele frequency ≥20% in tissue biopsies could also be detected in cfDNA, with >90% sensitivity and approximately 100% specificity (58). In contrast, Rivas et al. reported a sensitivity of detecting DLBCL mutations in cfDNA of 68% (59), whereas Liu et al. reported a sensitivity of 87.5% (60). The research by Rivas and Bohers both showed that the mutational landscape in cfDNA samples was highly consistent with that in biopsied tissue (59, 61). In a study of NHL, the genetic analysis of plasma cfDNA found that when detecting mutations that were detected at frequencies of greater than 20% in biopsy tissue, the sensitivity was 88.0%, and the specificity was greater than 99% (62). The consistencies of gene mutations detected among ctDNA and biopsied tissues from extranodal NK/T-cell lymphoma (nasal type), T-cell lymphoblastic lymphoma, and angioimmunoblastic T-cell lymphoma were 93.75%, 89.1%, and 83%, respectively (63–65). The above results indicated that cfDNA/ctDNA could be used to assess the tumor genotypes of NHL patients as alternative methods for tissue biopsy. In addition, Rossi et al. confirmed that mutations could be identified in cfDNA that were undetectable in tissue biopsies due to the spatial heterogeneity of the tumor (58). Therefore, cfDNA can be used as a supplementary test for cell-of-origin (COO) classification. Scherer et al. used NGS to detect ctDNA from pretreatment plasma for COO typing in DLBCL, and the results were 80% consistent with those derived using the Hans algorithm (66). They concluded that ctDNA genotyping could be used to classify DLBCL based on COO analysis (66). Sun et al. found that the mean concentration of cfDNA was significantly higher in non-GCB patients than that in GCB patients (15.95 ng/ml vs. 4.72ng/ml, p = 0.015) (37).

The above research results show that cfDNA can be used for genotyping analysis, providing a more straightforward and easier detection method for determining disease classification criteria in NHL. Although cfDNA can compensate for the heterogeneity associated with tissue biopsy and provide more supplementary information regarding genetic phenotypes, current research has indicated differences between cfDNA and tumor biopsies for the analysis of phenotypic results. No evidence has supported the use of cfDNA to completely replace biopsies for tumor genotyping.

Unfortunately, the results of studies examining PCNSL have not been as promising as those examining systemic DLBCL. Hattori et al. detected cfDNA evaluated by droplet digital PCR in 14 patients, among whom the *MYD88 L265P* mutation was identified in tumor-derived DNA. The *MYD88 L265P* mutation was detected in only eight of the 14 cfDNA samples (67). Montesinos et al. also detected *CD79b* and *MYD88* in both tumor-derived DNA and cfDNA. Among 27 PCNSL tissue biopsies, *CD79B* and *MYD88* mutations were detected in 59% and 85% of cases, respectively; however, the detection rates for the *CD79B* and *MYD88* mutations in cfDNA from the same group of patients were only 0% and 4%, respectively (68). These results showed that in PCNSL, even if the patient carries the *CD79B* or *MYD88* mutation, these mutations may not be detectable in blood-derived cfDNA (68). These studies indicate that unlike systemic DLBCL, circulating cfDNA may not be applicable for the genotyping analysis of PCNSL. Montesinos et al. proposed that the detection of genetic mutations in cfDNA may indicate the possibility of systemic DLBCL and distinguish PCNSL and second central nervous system lymphoma (68).

## LBs as Biomarkers for Prognosis

A number of studies have shown that the levels of cfDNA/ctDNA may be associated with poor clinical prognosis indicators, and at least two studies have reported that the levels of cfDNA/ctDNA were significantly associated with age, B symptoms (34, 35), disease stage (34, 35, 59, 66), and the International Prognostic Index (IPI) score (34, 35, 37, 59, 61) in DLBCL. In T cell lymphoblastic lymphoma, studies have confirmed that ctDNA concentrations are associated with IPI scores (64). These indicators are highly correlated with poor prognosis. However, whether circulating DNA levels are related to lactate dehydrogenase (LDH) levels remains controversial. LDH is considered to be an indicator of tumor burden of lymphoma. Rivas et al. (59), Sun et al. (37), and Bohers et al. (61) have shown that the detection of cfDNA/ctDNA in DLBCL is related to LDH levels. In contrast, Hur et al. (33) and Eskandari et al. (34) found no strong correlations between serum LDH and cfDNA in DLBCL. The relationship between cfDNA and Ki-67 is also unclear. Only Wu et al. have examined the relationship between cfDNA and Ki-67 in NHL, and they found that the concentration and integrity of cfDNA were significantly correlated with Ki-67 expression (69). The study also confirmed that cfDNA concentration and integrity are significantly related to the Eastern Cooperative Oncology Group (ECOG) score, LDH level, disease stage, IPI score, and B symptoms (69). In addition, higher concentrations of ctDNA have been significantly correlated with total metabolic tumor volumes, assessed by positron emission tomography/computerized tomography (PET/CT), in both DLBCL and extranodal NK/T-cell lymphoma (59, 63, 66, 70), indicating that ctDNA levels might serve as a surrogate for tumor burden. Many studies have confirmed that high levels of cfDNA/ctDNA in NHL, including DLBCL, FL, TCL, and NKTCL, are associated with shorter progression-free survival (PFS) and overall survival (OS) (33, 34, 36, 59, 71, 72), which indicates that high cfDNA/ctDNA concentrations are poor prognostic factors for NHL. Other studies have examined the DNA methylation patterns in cfDNA. Kristensen et al. analyzed 158 plasma samples from DLBCL patients and examined the DNA methylation patterns in the promoter regions for death-associated protein kinase 1 (*DAPK1*), deleted in breast cancer 1 (*DBC1*), *MIR34A*, and *MIR34B*/*C* using pyrosequencing (20). They concluded that *DAPK1* methylation was an independent prognostic factor for OS (P < 0.0007) (20). Chiu et al. profiled genome-wide 5-hydroxymethylcytosine (5hmC) patterns in plasma cfDNA from 48 DLBCL patients and found that the cfDNA 5hmC profiles were associated with the disease stage and IPI score (73). These results indicated that DNA methylation in cfDNA at diagnosis might also be associated with prognosis, representing a new predictive strategy for DLBCL.

A large number of favorable results have indicated that cfDNA/ctDNA have great potential for prognosis. However, no new prognostic model has been developed based on cfDNA/ctDNA. Whether cfDNA/ctDNA can serve as independent prognostic indicators of NHL and the determination of the prognostic efficacies and accuracies of cfDNA/ctDNA will require further studies.

A large number of circulating miRNAs have been associated with DLBCL; therefore, researchers are also exploring the value of miRNAs for prognosis. In 2008, Lawrie et al. found that high miR-21 expression levels were associated with longer relapse-free survival in DLBCL (40), and Chen et al. reached a similar conclusion (39). Conversely, upregulated miR-21 has been described as an independent and poor prognostic factor in another study (51), and the increased expression of miRNAs is thought to be related to poor prognosis. A high expression level of serum miR-22 in DLBCL at diagnosis is an independent prognostic factor for estimating PFS (74). DLBCL patients with upregulated miR-155 (41) and miR-125b (46) had shorter OS. Plasma high levels of miR-20a/b, miR-93, and miR-106a/b were associated with higher mortality in DLBCL (47). Although a variety of prognostic-related miRNAs have been identified, their prognostic efficacy and whether they can be applied to a prognostic model for the accurate stratification of patients remains unclear. Recently, a prognostic model for DLBCL based on four circulating miRNAs (miR21, miR130b, miR155, and miR28) was established and tested in a training cohort of 279 patients and a validation cohort of 225 patients (NCT01852435) (75). However, the potential application of this model requires additional clinical data. We also expect that any existing prognostic models for DLBCL and other NHL subtypes will likely improve as more miRNA research results become available.

Although few studies have examined the relationship between exosomes and prognosis, the results of several published studies have shown that the concentrations of TDEs and the miRNA or protein components contained in TDEs have good prognostic potential. The research by Feng et al. examining exosome miRNA in 2019 showed that the increased levels of miR-99a-5p and miR-125b-5p in serum exosomes derived from DLBCL patients were associated with shorter PFS (76). In 2020, the same team found that the increased expression level of carbonic anhydrase 1 (CA1) in serum exosomes from DLBCL patients was associated with inferior PFS and IPI scores (77). Zare et al. observed that refractory/relapsed DLBCL patients receiving R-CHOP therapy presented with higher concentrations of exosomes and exosomal miR-155 levels than responsive patients (78). Therefore, they speculated that exosome miR-155 might be used as a potential biomarker for predicting the response of DLBCL patients to treatment (78). Ryu et al. found that in extranodal NK/T-cell lymphoma, high levels of miR-4454, miR-21-5p, and miR-320e in serum exosomes were significantly related to poor OS (79). As the roles of large numbers of miRNAs are gradually being unraveled, the prognostic roles of circulating exosomes require additional exploration. With the deepening of research, the prognostic potential of TDEs will gradually become more apparent.

## LBs in Treatment

cfDNA can be used to detect a variety of gene mutations and abnormal pathways, and these changes can be developed as therapeutic targets. Therefore, many researchers suspect that testing cfDNA at diagnosis may be able to guide subsequent treatment. Camus et al. used digital PCR to detect *XPO1*, *MYD88*, and *EZH2* mutations in cfDNA from DLBCL patients (80). Hayashida et al. found that angioimmunoblastic T-cell lymphoma patients carried *RHOA^G17V^* and *IDH2^R172^* mutations in cfDNA (81). These related mutations may be beneficial for guiding the selection of targeted drugs. However, no studies have confirmed whether the development of treatment strategies based on cfDNA can benefit patients.

Similar to cfDNA, miRNA has been shown to be associated with some gene mutations and signaling pathways. In 2017, Beheshti et al. identified ten circulating miRNAs in a DLBCL mouse model (let-7b, let-7c, miR-10b, miR-130a, miR-155, miR27a, miR-24, miR-18a, miR15a, and miR-497) that strongly impacted JUN and MYC oncogenic signaling (53). Khare et al. identified mRNAs that were targeted by abnormally expressed miRNAs (miR-124, miR-532-5p, miR-141, miR-145, miR-197, miR-345, miR-424, miR-128, and miR-122) in the DLBCL model mice and the biological processes in which they were involved (47). The results showed that these miRNAs might upregulate signal transducer and activator of transcription 3 (STAT3), interleukin 8 (IL8), phosphoinositide 3-kinase (PI3K)/protein kinase B (AKT), and transforming growth factor (TGF)-β signaling pathways and downregulated the phosphatase and tensin homolog (PTEN) and p53 signaling pathways (47). In addition, Cui et al. found that miR-494 was differentially upregulated in immunosuppressive monocytes and macrophages, and the levels of miR-494 and miR-21 decreased within 3–6 months after DLBCL patients initiated immunochemotherapy (38). In B lymphoma cells and DLBCL patients, miRNAs (miR-21, miR-130b, miR-155, and miR-28) were found to regulate Ras signal transduction through insulin-like growth factor (IGF1) and JUN, participating in the induction of myeloid suppressor cells and Th17 cells (75). These results suggested that miRNA expression and detection may be able to guide treatment, representing a novel treatment strategy.

Compared with circulating nucleic acids, exosomes represent a critical component of the malignant tumor microenvironment, involved in tumor progression, metastasis, immune escape, and other factors, and may have tremendous therapeutic potential. B lymphoma cell-derived exosomes contain proteins that are involved in antigen presentation, cell migration, and cell adhesion and have been shown to carry tumor surface antigens, such as CD19, CD20, and CD22 (82). These results indicated that B lymphoma cell-derived exosomes might be able to induce tumor antigen-specific anti-tumor immunity. *In vivo* experiments in a mouse model of TCL also confirmed that TDE could induce humoral and cellular immune responses (83). Therefore, TDE may serve as a potential source of lymphoma cell-related antigen immunotherapy. Chen et al. demonstrated that exosomes from DLBCL cell lines express tumor-related molecules, including c-Myc, Bcl-2, Mcl-1, CD19, and CD20 (84). TDEs from DLBCL can be captured by dendritic cells and lymphoma cells and exert an immunosuppressive effect by inducing T cell apoptosis and the upregulation of programmed cell death protein 1 (PD-1) (84). After being pulsed with TDEs, dendritic cells might stimulate the clonal expansion of T cells, increasing the secretion of IL-6 and tumor necrosis factor α (TNFα), and decreasing the production of immunosuppressive cytokine IL-4 and IL-10 (84). This study provides a theoretical basis for TDE to become a treatment for DLBCL. These results show that TDE can be used as a new immunotherapy target for NHL and the use of exosome-derived vaccines to enhance anti-tumor immune responses. Exosomes can protect cargo from nucleases and proteases during cell communication, have low immunogenicity and cytotoxicity, and have the ability to target tumor cells specifically; therefore, they are often regarded as molecular signaling factors and have been examined as carriers in applications such as drug delivery and horizontal gene transfer (85). Exosomes derived from malignant tumors can promote immune escape and directly or indirectly support tumor cells in the tumor microenvironment. Blocking the secretion of exosomes from tumor cells or removing them from the patient’s body may inhibit tumor progression, suggesting that targeting TDEs may represent a novel strategy for NHL treatment.

Immunochemotherapy has achieved remarkable results in the treatment of NHL, and a variety of immunotherapies based on molecules in B-cell NHL surface provide many new possibilities for improving the prognosis of NHL (86). LBs, which can be used as a biomarker, a therapeutic target or a screening tool for immunotherapy, plays an important role in a variety of new immunotherapies such as immune checkpoints, bispecific antibodies, and antibody-drug conjugates (86).

In addition, LBs may hold great promise with tumor microenvironment. The tumor microenvironment provides protection for tumor cells and suppresses the immune response. LBs may be used to monitor and regulate the tumor microenvironment in NHL. As we all know, angiogenesis plays a key role in the progression and prognosis of NHL (87). Angiogenesis provides tumors with sufficient oxygen, nutrients and an effective metabolic waste removal system (88). Angiogenesis also provides an “escape route” for tumor cells, thereby promoting tumor spread and metastasis (88). miRNA is a regulator of tumor angiogenesis (87). It can not only play an anti-angiogenesis effect, but also act as a pro-angiogenesis factor (87). Therefore, miRNA has great potential in regulating tumor microenvironment and improving the treatment response of NHL.

## LBs as Biomarkers to Evaluate Response to Treatment

Sidaway et al. used ctDNA to assess the early response of DLBCL patients to treatment (89). Within one week of starting treatment, the ctDNA levels of responders were significantly reduced, allowing the responders and non-responders to be fully distinguished before the end of the first treatment cycle (89). The 24-month event-free survival (EFS) of patients who displayed early molecular response (EMR) and major molecular response (MMR) were significantly improved compared with other patients, and EMR patients who ultimately required rescue treatment also showed higher 24-month EFS (89). Dynamic changes in cfDNA/ctDNA levels can provide early indications of clinical outcomes (89), although the results of cfDNA/ctDNA research have been controversial. A study of NHL compared cfDNA at the time of diagnosis with various time points during the treatment period (69). The results showed that the concentration and integrity of cfDNA during the diagnosis stage were significantly higher than those during the treatment stage (69). By comparing and analyzing the plasma cfDNA of DLBCL patients after R-CHOP treatment, LDBCL-related mutations were quickly cleared in patients who responded to treatment, and the basic mutations in cfDNA did not disappear from those patients who were drug-resistant (58). In addition, in drug-resistant patients, new mutations were detected in cfDNA, and resistant clones were screened during the process of clonal evolution (58). In contrast, a recent study by Hur et al. reached a different conclusion (33). They analyzed the cfDNA from DLBCL patients before and during various chemotherapy regimens and found no significant differences in PFS and OS regardless of the cfDNA concentration (33). Changes in cfDNA levels before and after treatment may be caused not only by changes in tumor burden but also by other conditions, including infection or chemotherapy-induced inflammation (33). These discrepant findings indicated that some uncertainty exists regarding the use of cfDNA concentration to monitor treatment response. In addition, cfDNA has also been reported to be highly expressed in conditions other than malignant tumors, such as inflammation and infection. After patients receive chemotherapy, the effects of cytotoxic drugs are likely to result in inflammation-related changes in the microenvironment. Due to the small number of studies, whether cfDNA concentration can be used to monitor early treatment response remains unclear. In addition, whether any changes in cfDNA observed during treatment is related to the patient’s prognosis remains controversial. The use of cfDNA concentration as an indicator of efficacy in the monitoring and guidance of treatment requires careful consideration.

A study analyzed 736 miRNAs in the serum of 20 patients with complete DLBCL remission and patients with primary refractory DLBCL (90). Five miRNAs (miR-224, miR-1236, miR-520d-3p, miR-33a, and miR-455-3p) were differentially expressed between the two groups and were verified in other 133 patients (90). The upregulation of miR-455-3p and miR-33a was associated with chemosensitivity, whereas the upregulation of miR-224, miR-1236, and miR-520d-3p was associated with chemoresistance (90). Other studies reported that miR-22, miR-494, and miR-21 were downregulated in DLBCL after immunochemotherapy (38, 74). miR-494 and miR-21 expression was reduced in patients who were PET/CT-negative but not in those who remained PET/CT-positive (38). In DLBCL patients who did not respond to treatment, plasma miR-21 and miR-197 levels were significantly upregulated (50). The circulating levels of miR-125b and miR-130a may be related to R-CHOP resistance (46). Overall, these results indicated that differences in circulating miRNA expression before and after treatment might reflect the treatment response or disease progression. Therefore, circulating miRNAs have the strong potential to serve as predictive and monitoring indicators of treatment response in DLBCL patients. However, only miR-21 has been shown to be significantly associated with treatment response in more than one study. More research remains necessary to obtain a large amount of reliable data.

With the discovery of TDEs in DLBCL patients in recent years, researchers have also explored the possibility of using exosomes to monitor tumor progression. Xiao et al. found that the expression level of exosomal miR-451a in the DLBCL group was significantly lower than that of the control group (56). After treatment with rituximab combined with chemotherapy, the level of serum exosomal miR-451a in treated patients was significantly increased, although it remained below the normal level (56). Feng et al. found that the expression level of CA1 in exosomes was significantly increased in drug-resistant DLBCL cells compared with drug-sensitive DLBCL cells, and the presence of CA1 in exosomes can boost chemotherapy resistance *via* the nuclear factor (NF)-κB and STAT3 pathways (77). These results suggested that TDE could be used to evaluate treatment response. However, additional research remains necessary to explore and verify this issue.

## LBs as Biomarkers in Disease Monitoring

Imaging is currently the primary method used for detecting NHL. However, imaging has many limitations. CT has low sensitivity for disease detection, and although the use of PET/CT can improve sensitivity, PET/CT still has low tumor specificity and a high false-positive rate (91). More importantly, imaging cannot directly assess diseases at the molecular level or dynamically monitor or identify the biological mechanisms that drive the development of tumors, such as tumor heterogeneity and clonal evolution. In addition, imaging tests are associated with other risks, including radiation exposure, the use of invasive imaging agents, and high cost. A meta-analysis of 737 patients with DLBCL showed that a considerable number of patients relapsed during follow-up (91). The commonly used PET/CT protocols have a spatial resolution of 6–7 mm, which cannot detect minimal residual disease (MRD), which is a common origin of recurrence (18, 92).

The concept of MRD is an excellent surrogate for the evaluation of curative effects, the guidance of treatment, and the prediction of outcomes. MRD has been widely used in a variety of hematological tumors, including acute myeloid leukemia (AML) and acute lymphocytic leukemia (ALL) (93, 94). However, NHL patients typically lack leukemic involvement; therefore, the detection of MRD in NHL using current conventional methods is not currently possible. Fortunately, the discovery of circulating nucleic acids and circulating exosomes have introduced the possibility of evaluating MRD at the molecular level. MRD can be detected in the blood by studying tumor mutation using a variety of techniques, including PCR–based methods, and NGS–based techniques (21).

Scherer et al. followed up and monitored the ctDNA levels of 11 DLBCL patients and found that in 8 (73%) patients, ctDNA could be detected as an indicator of MRD before disease relapse (66). The average time from the discovery of ctDNA to clinical relapse was 188 days (66). In a National Cancer Institute (NCI) study, the ctDNA of 17 patients with DLBCL who progressed after complete remission (CR) was analyzed, and 15 were found to be positive for ctDNA before progression was detected (95). Chen et al. monitored the ctDNA of 14 T cell lymphoblastic lymphoma patients after treatment and found that in the 2 cases that relapsed during the maintenance phase, ctDNA could be detected before recurrence (64). The time from the detection of ctDNA to clinical recurrence was 83 and 84 days in these patients (64). Shin et al. followed up the ctDNA levels of NHL patients after treatment and found that 93% (13 of 14) of patients who achieved CR presented declining ctDNA, and ctDNA disappeared in most patients. In the 2 cases in which trace ctDNA could be detected, the disease progressed after 6 months of ctDNA monitoring (62). These results indicated that ctDNA could be used as an indicator to monitor recurrence or disease progression. However, Suehare et al. suggested that the *TP53* and *DNMT3A* mutations detected in the cfDNA of DLBCL patients after the disease remission could be derived from clonal hematopoiesis of indeterminate potential (CHIP) rather than from MRD (96). The results of LBs should be analyzed more rigorously, especially for those gene mutations that are shared between lymphoma and CHIP (96). The source of cfDNA is more complicated than that of ctDNA. However, for cfDNA/ctDNA, the current research results are minimal, and more prospective research remains necessary.

Unfortunately, the results of a study conducted in PCNSL were not satisfactory. Hattori et al. detected the *MYD88 L265P* mutation in cfDNA during and after chemotherapy treatments in five patients with PCNSL, among whom the *MYD88 L265P* mutation was detected in cfDNA at diagnosis (67). However, they found that the *MYD88 L265P* mutation was not detected in cfDNA even if the disease progressed (67), which may indicate that *MYD88 L265P* may be negative in relapsed patients, or cfDNA might not circulate in the peripheral blood after chemotherapy (67). These findings indicated that cfDNA might not be applicable for the monitoring of MRD in PCNSL. However, the study only analyzed five patients, and the role of cfDNA for monitoring the MRD of PCNSL requires additional research.

Although few studies have examined the use of miRNA as a monitoring indicator, they all confirmed that miRNA has the potential for use in the monitoring of MRD or progression and recurrence. Among DLBCL patients with CR, high levels of miR-19b, miR-20a, and miR-451 were able to distinguish MRD-positive patients from patients without residual disease (50). In addition, studies have found that changes in the serum miR-130a and miR-125b levels of DLBCL patients are detectable in the clinical diagnosis of relapse or progression (46).

## Discussion

The evaluation of circulating DNA, cfDNA/ctDNA, as a type of LB has been increasingly studied. LBs can play vital roles in the diagnosis, classification, prognosis, and treatment of NHL. During the diagnosis and classification of NHL, tissue biopsy continues to play a decisive role; however, the evaluation of circulating DNA can be used as a complementary detection method. Tissue biopsy is difficult to use to detect recurrence or evaluate MRD because biopsy is invasive and cannot be repeated multiple times. Imaging has limited sensitivity and cannot detect changes that occur at the molecular level. The advantages of evaluating circulating DNA were discussed in-depth, and research has revealed the developmental potential for circulating DNA both during and after treatment. However, the use of cfDNA/ctDNA as a surrogate marker requires the optimization and standardization of pre-analytical steps and analysis techniques.

Researchers have similar expectations regarding the evaluation of another circulating nucleic acid: miRNA. However, although a large number of miRNAs have been studied, only miR-21 has obtained relatively consistent results in multiple studies, suggesting that it may play a role in diagnosis, prognosis, and response assessment.

Circulating exosomes contain a great deal of information, enabling a more comprehensive understanding of disordered signal transduction processes and the expression of related antigens for diagnosis and treatment. In contrast, circulating nucleic acids cannot provide information about changes in the proteome and transcriptome of lymphoma. Although few relevant studies have been published, these studies have confirmed the potential role of TDEs in the diagnosis, prognosis, treatment, and monitoring of NHL. In particular, the physiological properties of circulating exosomes make them more potential options for improved treatment.

Very few studies have examined the use of peripheral blood for the performance of LB in PCNSL. The results of several cfDNA/ctDNA studies have not been satisfactory thus far. Compared with cfDNA/ctDNA in peripheral blood, CSF ctDNA seems to have more advantages in the management of PCNSL. CSF ctDNA can better detect central nervous system lymphoma than plasma ctDNA (97). Analysis of gene mutations such as CD79B and MYD88 in CSF can be used as a molecular diagnostic method for PCNSL (10). The longitudinal analysis of CSF ctDNA revealed that sustained tumor responses were associated with the clearance of ctDNA from the CSF (98, 99). CSF ctDNA predicted central nervous system relapse in central nervous system and systemic lymphomas (97). Research on circulating exosomes in PCNSL is almost nonexistent. PCNSL is a malignant tumor that originates in the brain, making the acquisition of tissues during diagnosis or after treatment particularly difficult and enhancing the developmental prospects for LB in PCNSL. More than 90% of PCNSL pathology is DLBCL, which provides a theoretical basis for extending the LB results associated with DLBCL to PCNSL.

Although LB has been associated with positive findings in many aspects of the disease, some questions remain worth considering. (1) Thus far, no widely accepted standard protocols have been developed for the collection, processing, and storage of samples, which may lead to pre-analysis bias and varying results. (2) The best time and duration for monitoring remains undetermined. (3) cfDNA is dysregulated in many disease states, and no reliable standard has been established to distinguish normal exosomes from TDEs, which can make the interpretation of results difficult. (4) The detection cutoff levels set by different groups have been inconsistent, resulting in a lack of comparability among the results. (5) The current research primarily focuses on DLBCL, with little data and results regarding other NHL subtypes. (6) A large number of reasonably designed prospective studies remain necessary to verify the reported findings. (7) A better understanding of the biological characteristics of circulating nucleic acids and exosomes will help optimize their use.

## Conclusions

The noninvasive, easy-to-obtain, and reproducible characteristics of LB provide unique opportunities for the dynamic assessment of NHL-related changes. This method improves the diagnostic accuracy of identifying disease subtypes and prognosis and can be used to provide better-individualized treatment for patients. More importantly, LB can be used to fill the gaps in the monitoring of response to treatment and the detection of MRD in NHL. The real-time monitoring of treatment and timely adjustments to treatment plans based on identifiable characteristics will provide enhanced benefits to patients. The accurate early detection of disease recurrence can also enable clinicians to perform remedial treatments earlier. In-depth research examining the various substances in the circulatory systems of cancer patients will also help clarify the pathogenesis of NHL and promote the development of new treatment strategies.

## Author Contributions

LL wrote the manuscript. YL reviewed and edited the manuscript in detail. All authors contributed to the article and approved the submitted version.

## Funding

This work was supported by Capital’s Funds for Health Improvement and Research, NO.2020-2-2049.

## Conflict of Interest

The authors declare that the research was conducted in the absence of any commercial or financial relationships that could be construed as a potential conflict of interest.
